# Audit of Admission Blood Tests and Vitamin D Pathway Adherence Amongst New Admissions to an Inpatient Psychiatry Unit

**DOI:** 10.1192/bjo.2026.11741

**Published:** 2026-06-30

**Authors:** Lydia Brown, Keith Ngwenya

**Affiliations:** South West Yorkshire Partnership NHS Foundation Trust, Barnsley, United Kingdom

## Abstract

**Aims:**

This audit aims to assess adherence to RCPsych guidance regarding timely admission blood tests (performed within 24 hours of admission), and what blood tests were performed as part of this. In addition, we aimed to evaluate adherence to Trust policy regarding vitamin D and calcium assessment and appropriate management of deficiency.

**Methods:**

Retrospective case audit evaluating admissions to an inpatient adult working age psychiatry unit in December 2025 (excluding PICU patients). Records of 19 patients were reviewed (10 female, 9 male). Clinical notes and ICE records were used to obtain data. Information gathered included date of admission, date and results of admission blood tests, documentation of these blood results on the clinical system and medication prescriptions and discharge letter details.

**Results:**

Of 19 patients, 79% had bloods done within 24 hours of admission. For 1 patient, these samples were in adequate and so a repeat sample was treated as their admission set for purposes of this audit. Of those with a delay >24hrs, 75% had a reason documented.

The most common admission bloods performed were full blood count, urea & electrolytes and liver function tests (84%), followed by thyroid function tests and haematinics (79%), lipid and calcium profiles (74%), HbA1c and prolactin (63%), with glucose done least commonly (53%). Vitamin D levels were checked in 68% of patients (including 1 patient who did not initially have vitamin D levels checked, but levels were subsequently done).

Of those with a reported vitamin D level, 23% had sufficient levels, 31% had mild deficiency, and 54% had significant deficiency.

Of those with significant deficiency, all had vitamin D loading doses prescribed. However, only 50% of those with mild deficiency had vitamin D prescribed. At the point of data collection, 26% of the patients involved in this audit had been discharged; 67% of those discharged had a significant vitamin D deficiency, but only 75% of these patients had ongoing supplementation provided. None of these patients had a plan to re-check serum calcium as per guidelines on their discharge letter.

**Conclusion:**

In conclusion, although the majority of new inpatient admissions had some form of bloods taken within 24hrs of admission, the type of bloods they had done was variable. Vitamin D levels were checked in 74% of patients, with 50% of those having a significant vitamin D deficiency. Initial prescription of loading dose therapy had a high compliance but follow up appeared poor, representing an area of possible improvement.

